# DIPEND: An Open-Source Pipeline to Generate Ensembles of Disordered Segments Using Neighbor-Dependent Backbone Preferences

**DOI:** 10.3390/biom11101505

**Published:** 2021-10-12

**Authors:** Zita Harmat, Dániel Dudola, Zoltán Gáspári

**Affiliations:** 1 Faculty of Information Technology and Bionics, Pázmány Péter Catholic University, Práter Str. 50/A, 1083 Budapest, Hungary; harmat.zita@itk.ppke.hu (Z.H.); daniel.dudola@gmail.com (D.D.); 2 3in Research Group, Faculty of Information Technology and Bionics, Pázmány Péter Catholic University, 2500 Esztergom, Hungary

**Keywords:** protein ensemble model, intrinsically disordered proteins, structure prediction, principal component analysis, local interaction, dihedral angle

## Abstract

Ensemble-based structural modeling of flexible protein segments such as intrinsically disordered regions is a complex task often solved by selection of conformers from an initial pool based on their conformity to experimental data. However, the properties of the conformational pool are crucial, as the sampling of the conformational space should be sufficient and, in the optimal case, relatively uniform. In other words, the ideal sampling is both efficient and exhaustive. To achieve this, specialized tools are usually necessary, which might not be maintained in the long term, available on all platforms or flexible enough to be tweaked to individual needs. Here, we present an open-source and extendable pipeline to generate initial protein structure pools for use with selection-based tools to obtain ensemble models of flexible protein segments. Our method is implemented in Python and uses ChimeraX, Scwrl4, Gromacs and neighbor-dependent backbone distributions compiled and published previously by the Dunbrack lab. All these tools and data are publicly available and maintained. Our basic premise is that by using residue-specific, neighbor-dependent Ramachandran distributions, we can enhance the efficient exploration of the relevant region of the conformational space. We have also provided a straightforward way to bias the sampling towards specific conformations for selected residues by combining different conformational distributions. This allows the consideration of a priori known conformational preferences such as in the case of preformed structural elements. The open-source and modular nature of the pipeline allows easy adaptation for specific problems. We tested the pipeline on an intrinsically disordered segment of the protein Cd3ϵ and also a single-alpha helical (SAH) region by generating conformational pools and selecting ensembles matching experimental data using the CoNSEnsX${}^{+}$ server.

## 1. Introduction

### 1.1. Ensemble-Based Modeling of Protein Internal Dynamics

Despite significant developments in protein structure prediction and modeling such as AlphaFold2 [1], the detailed description and interpretation of protein internal motions is still a considerable challenge. One of the most efficient methods is to generate structural ensembles that reflect experimentally determined parameters, obtained typically from NMR and/or SAXS measurements [2,3]. The number of such ensembles is growing as reflected in the recently updated Protein Ensemble Database [4,5]. The distinctive feature of such ensembles is that they reflect the experimentally determined parameters as a whole, and no individual conformer is expected to correspond to all the data. This is based on the rationale that the measured data correspond to a time- and ensemble-average themselves, and it might not even be realistic that a single conformation can be described by these parameters. Such ensembles can typically be generated by restrained molecular dynamics simulations or selection-based approaches, where a subset of conformers is chosen from a large pool. The former one can be primarily used for systems that are expected to fluctuate around a more or less well-defined average state such as in the case of fast motions described by $S^{2}$ order parameters or Residual Dipolar Couplings (RDCs) [6,7,8,9]. Besides the relatively limited conformational space explored, the other potential drawback of restrained MD is that the restraining forces might distort the force field and might result in unrealistic geometry.

Pool-based selection approaches can avoid both of these pitfalls and are applicable to molecules adopting highly diverse structures in the course of their fluctuations. In this case, however, the proper sampling of the conformational space is critical as it should focus on the relevant region which is not known a priori. Therefore, it is commonly accepted that for the modeling of intrinsically disordered segments, a very large number of structures, on the order of tens of thousands, should be generated [10]. For this purpose, a number of approaches have been developed that often include all steps from pool generation to selection. One of them is Flexible Meccano [11,12,13], which aims to build structures of IDPs from fragments of PDB-deposited disordered protein segments. Another approach to sample the most relevant region of the conformational space is to consider the effects of neighboring residues by applying a triplet-based conformer library [13]. The ENSEMBLE method [10] performs both the generation and selection steps, and accepts pre-generated additional ensembles to widen the conformational space explored by the TraDes method [14,15], used by default. Structure pools can also be obtained from suitably parametrized MD simulations [16]. In practice, adjustment of the conformational search space to sample structures with predefined local conformational preferences is often desirable to obtain ensembles with good correspondence to experimental data [17].

Our motivation was to add an open-source and easily configurable method to this continuously developing toolkit that can be used either alone or in combination with other approaches and is based on independently established conformational preferences of amino acids. To this end, we created an open-source solution that relies on software that is actively maintained and freely available, as well as on publicly available datasets. Our pipeline, called DIPEND (DIsordered Protein Ensembles from Neighbor-dependent Distributions) is implemented in Python3 and is available on GitHub https://github.com/PPKE-Bioinf/DIPEND. It uses ChimeraX [18], Scwrl4 [19] and Gromacs [20] as well as neighbor-dependent Ramachandran distributions available from the Dunbrack lab [21]. We have chosen this model because of its solid theoretical background, robustness and availability. The advantage of using ChimeraX and GROMACS is that these programs are widely used, free and actively maintained, and in the long run, the accessibility and flexibility of these methods, allowing adjustments by the users, outweighs the computational costs associated with this design. To enhance the sampling of conformations expected to be relevant for a given system, our implementation allows for the combination of neighbor-dependent distributions with user-defined ones with adjustable relative weighting. Thus, local structural preferences can explicitly be taken into account during ensemble generation on a per-residue basis.

Our design relies on decoupling ensemble generation from evaluation. The reason for this is that we believe that it provides a fully transparent pipeline and allows the user to choose the evaluation method independently. Here, the user intervention ensures that the bias introduced is justifiable and intentional by the researcher. The evaluation of the ensembles is often non-trivial as it depends both on the purpose of the modeling and the availability and reliability of the experimental data used for validation. In this description, we use our previously created CoNSEnsX${}^{+}$ server [22] extended with the analysis of secondary chemical shifts to demonstrate the application of local structural preferences in ensemble generation.

### 1.2. Neighbor-Dependent Ramachandran Preferences for Amino Acids

For DIPEND, we have chosen to use the dataset compiled by Ting et al. [21]. They have calculated probabilities for the different ϕ and ψ dihedral angles of a particular amino acid having a specific amino acid as “left” (N terminal side sequential) neighbor or “right” (C terminal side sequential) neighbor. To obtain a smooth distribution, they fitted their data to a hierarchical Dirichlet model. Thus, the reported backbone dihedral probabilities are a mixed sum of Gaussian probabilities. They explicitly consider *cis* and *trans* prolines as different residue types for the “central” amino acid investigated, but these are not considered separately as (“left” or “right”) neighbors.

### 1.3. Benchmark Test Systems

In order to assess our method, we have chosen two proteins with different structural preferences and availability of NMR parameters. The cytosolic domain of Cd3ϵ is an intrinsically disordered segment with no a priori known local structural preferences. In contrast, the single α-helix (SAH) domain of myosin VI exhibits a strong helical propensity. Therefore, these proteins represent suitable examples for testing the usability of the DIPEND pipeline.

#### 1.3.1. Cd3ϵ Cytoplasmic Domain

The Cd3ϵ (or T cell antigen receptor TCR) protein is part of the Major Histocompatibility Complex (MHC), a T cell receptor complex. Activation of this complex by an antigen (a peptide from a pathogen brought to the receptor by an antigen-presenting T cell) leads to a complex signal transduction cascade. This comprises phosphorylation of the complex at multiple sites, triggering the activation of several transduction pathways (such as the RAS, P53 and NFAT pathways), ultimately leading to T-lymphocite cell activation and immune response, such as the generation of effector T cells. It also has a role in calcium-induced signaling [23].

The cytoplasmic domain of the Cd3ϵ protein is largely intrinsically disordered and has been characterized by solution NMR spectroscopy [24]. We have investigated the disordered C terminal part, corresponding to residues 153–207 of the human Cd3ϵ protein containing an immunoreceptor tyrosine-based activation motif (ITAM). ITAMs are composed of two instances of the consensus sequence Y-X-X-[LI] separated by 6–8 residues [25]. We modeled the sequence as it appears in the corresponding BMRB entry (BMRB ID 18889 [24]) having serine and leucine as the first two amino acids instead of tryptophan and serine. We used the deposited chemical shift data for analysis.

#### 1.3.2. The α Helical Medial Tail Domain of the Porcine Myosin VI Protein

For modeling protein segments with a priori known structural preferences but flexibility not yet explicitly modeled with all-atom ensemble models, the α helical part of the medial tail domain of the myosin VI protein is an excellent example. This helical part serves as a lever arm to the myosin motor protein, which has a role in muscle contraction. For this α-helical region, a diverse set of NMR parameters was determined by Barnes et al., including ${}^{15}N$, ${}^{13}C$ and ${}^{1}H$ chemical shifts; ${}^{3}J_{HNHA}$ scalar couplings; and N-H, C-H and C-N residual dipolar couplings, as well as $S^{2}$ order parameters. Using NOE and RDC data, the solution structure of the segment was also determined and deposited in the PDB with ID 6OBI [26].

## 2. Materials and Methods

### 2.1. A Pipeline to Build Three-Dimensional Ensemble Models

Our pipeline is designed to build a number of structural models of a selected non-globular segment of a protein. Its only mandatory input with no default value is the sequence of the segment to be generated.

**Figure 1:**
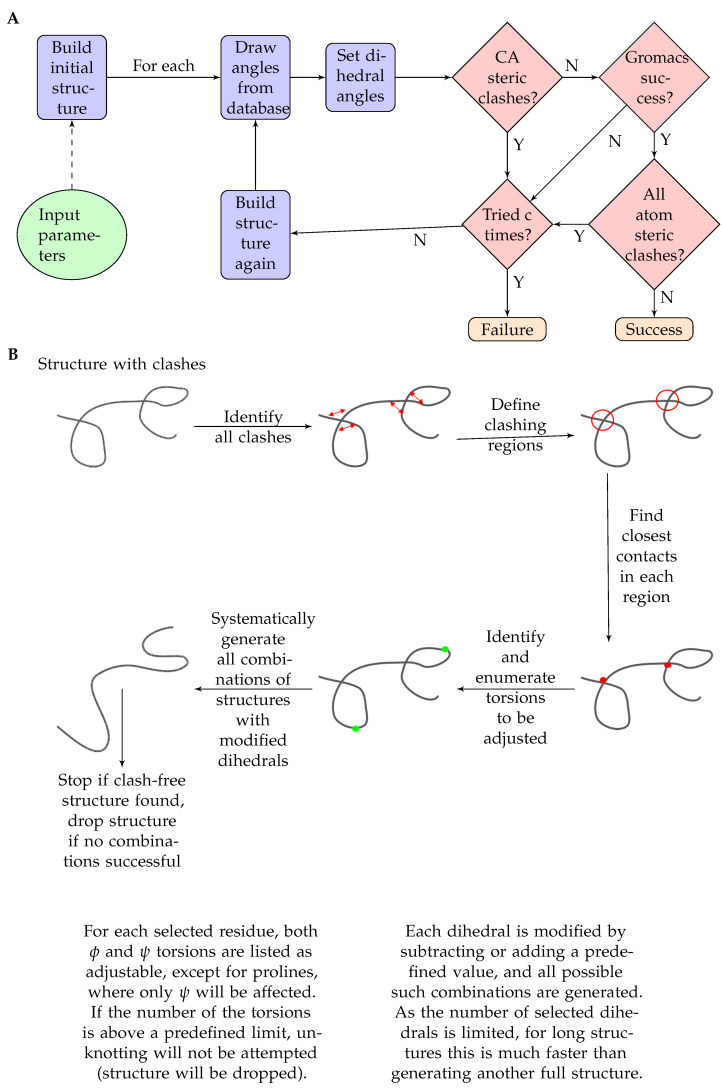
(**A**) Flowchart of the steps of the pipeline DIPEND (DIsordered Protein Ensembles from Neighbor-dependent Distributions). (**B**) Flowchart of the unknotting part of the DIPEND pipeline.

The working of the program is shown in (Figure 1). The main program is built in Python3.

The main steps performed by the program are:

- At first, ChimeraX [18] is invoked to build up a very long beta strand based on the given sequence.
- Next, Ramachandran angles for each residue are set based on the neighbor-dependent probabilities reported in [21]. The probabilities are defined for bins of 5x5 degrees and are stored as binary files to optimize speed and storage. Probabilities based on the left (N terminal side sequential) neighbor and right (C terminal side sequential) neighbor, as well as a combined one, derived as described in [21], are available and are denoted LEFT, RIGHT and TRIPLET. A roulette-wheel selection approach is used where a generated random number between 0 and 1 is used to select a bin according to the cumulative probabilities.
- ChimeraX [18] is again used to set the dihedral angles to the previously calculated values.
- For the obtained conformation, a quick check is performed to filter out unrealistic structures based on CA-CA distances below 4 Ångströms. This additional program is written in C++.
- If there are no CA steric clashes, the program Scwrl4 [19] is invoked to optimize the sidechains.
- If there are CA steric clashes, the program attempts to unknot them. If two residues clash, the program tries to perturb the dihedral angles of the residue halfway between them. The perturbation means to add or subtract a given value to the selected dihedral angles. For each angle, these perturbations are combinatorially applied, and the perturbed structures undergo the above clash check again.
- After that, the program runs GROMACS [27] to perform a short energy minimization in vacuum to optimize the structure. The generated log file is checked for success, as it is expected that structures with serious steric problems will fail this step.
- If the optimization is successful, ChimeraX [18] is invoked again to check for all atom steric clashes with its command clashes using default parameters. If there are no steric clashes, the structure is accepted successfully.
- The above steps are performed for each structure to be generated. For unsuccessful trials, the program will try and perform structure generation again until the user-defined limit for trials is reached or an accepted structure is generated.
- The input parameters of the program are:
  - The sequence (the only required input parameter);
  - The number of structures to be generated;
  - The building mode;
    * LEFT: considering only the left (N terminal) sequential neighbor in choosing dihedral angles;
    * RIGHT: considering only the right (C terminal) sequential neighbor in choosing dihedral angles;
    * TRIPLET: considers both sequential neighbors using derived cumulative probabilities;
    * WEIGHTED_LEFT: for each residue, a user defined Gaussian distribution can be combined with the Dunbrack distribution;
    * WEIGHTED_RIGHT: for each residue, a user defined Gaussian distribution can be combined with the Dunbrack distribution;
    * WEIGHTED_TRIPLET: for each residue, a user defined Gaussian distribution can be combined with the derived distribution.
  - a filename base for the generated structures (for example „bar_” as a base will result in bar_min_1.pdb, bar_min_2.pdb, etc.);
  - The dataset used (TCBIG or Coil only, see [21]);
  - The number of trials for a structure;
  - Whether a Gromacs optimization step should be performed for each structure;
  - Whether temporary files are kept after the run;
  - The angle to add or subtract from the dihedral angles at the unknotting steps;
  - The maximum number of torsions to be adjusted during unknotting, zero meaning no unknotting.

Some of the above described steps are further explained in the following subsections.

#### 2.1.1. The Initial Structure

As a first step, we build a long extended structure with the desired amino acid sequence using ChimeraX [18] with $\phi =-65^{\circ }$ and $\psi =+135^{\circ }.$

#### 2.1.2. Different Approaches to Handle Sequence Neighborhood

The next step is to apply the final dihedral angles for each amino acid based on its neighbor or assigned randomly. There are different building modes in our program, which means a different approach to handle sequential neighborhoods. The LEFT mode uses the cumulative probabilities reported in [21] to choose a 5-degree bin in the dihedral angles based on the left neighbor; the RIGHT mode is the same, but with the right neighbor, the TRIPLET mode uses the combination of the left and right probabilities as it is recommended by the Dunbrack lab. The calculation of the triplet probability is made by the following equation in the case of a cysteine as left neighbor and a glycine as right neighbor as an example:

(1) $$P_{triplet}=\frac{e^{ln\left(p_{cl}\right)+ln\left(p_{rg}\right)-ln\left(p_{lall}\right)-ln\left(p_{rall}\right)}}{p_{sum}}$$

where $p_{cl}$ is the left cysteine neighbor probability, $p_{rg}$ is the right glycine neighbor probability, $p_{lall}$ is the probability of all left neighbors, $p_{rall}$ is the probability of all right neighbors and $p_{sum}$ is the sum of all the different denominators in a given bin, therefore normalizing the probabilities. As a next step we evaluated this for the possible angle pairs and calculated the cumulative distribution by starting from (−180, −180) and for each angle pair adding the normalized respective probability to the previous sum. Normalization is simply dividing by the whole sum, so that at the last angle pair (+175, +175), it reaches exactly 1. The angles are eventually randomly drawn from these cumulative probabilities as described for the other building modes.

In the weighted modes, a Gaussian distribution can be defined for each residue. The central dihedral angles and the standard deviation can be set along with a weight which is between 0 and 1. For 0 weight, the user defined distribution is not considered, giving the same result as not using a weighted mode. At a weight of 1, only the user-defined Gaussian distribution is considered. For a given dihedral angle pair, the cumulative probability is derived as $p=w*p_{u}+\left(1-w\right)*p_{d}$, where w is the user defined weight, $p_{u}$ is the cumulative probability from the user-defined Gaussian distribution and $p_{d}$ is the cumulative probability of the Dunbrack data.

#### 2.1.3. Steric Clashes and Repetition

After the dihedral angles were selected with the given building mode, the angles were set individually for each amino acid with the program ChimeraX [18], calling it without GUI and graphics. The resulting structure is checked for steric clashes or discontinuities. If the distance between two CA atoms is less than 4 Ångströms and they are not sequential neighbors, it is considered a steric clash. If two CA atoms are closer than four amino acids in sequence and yet their distance is more than 20 Ångströms, this means that there is a discontinuity in the peptide chain. This latter case is not supposed to happen, but the former case is quite common, especially with a large structure more than 100 amino acids long.

Optionally, a so-called “unknotting” step can be performed for longer structures. The idea behind this is that for most clashes a minor adjustment of a few torsion angles might be sufficient to resolve the steric overlaps. The unknotting step is designed to provide only minimal deviation from the pre-selected backbone dihedral angles. First, all clashes are identified. Then, using clashes affecting neighboring residues, segments are defined, and for each segment, the closest inter-residue distance is kept along with the position of these clashing residues. In our heuristics, the residue to be adjusted to resolve this clash is the one in the middle of the segment defined by these two clashing residues. For each adjustable residue, both ϕ and ψ angles are considered, except for prolines, where only ψ will be adjusted. If the number of the torsions to be adjusted is above a predefined limit, no unknotting will be attempted, and the structure will be dropped. Otherwise, all combinations of these dihedrals will be explored by rotating each torsion in both directions with a predefined value (e.g., 30 degrees). Each of these structures will be checked for clashes, and the first clash-free structure will be accepted. If no such structure is found, the original structure will be dropped. Although the number of combinations to be explored during the unknotting step can be large, for long structures, this step is more efficient than generating a completely novel structure for each one with clashes. Both the maximum number of torsions and the extent of rotations can be set by the user.

The program keeps track of which structures are successfully built and which are not, based on other structure checks after performing an optional but highly recommended GROMACS minimization, and performs additional trials for the failed structures until the user-defined limit of tries is reached. For example, if we would like to make 10 structures and the number of tries is 30, in the worst case of an utter failure, there are 310 tries with no resulting correct structures, and in the very best case, there are 10 tries with 10 correct structures. We have used two datasets [21]: the coil only and the TCBIG, which contains all kinds of secondary structures, except alpha helical and beta strand.

#### 2.1.4. Selecting and Evaluating Subensembles

Subensemble selection was performed with a locally installed instance of the dockerized version of CoNSEnsX${}^{+}$ [22]. CoNSEnsX${}^{+}$ selects a subensemble which has the best correspondence to the experimental data. A greedy algorithm is used starting with a single structure which reflects the experimental data the most. After that, it adds other conformers step by step if it improves the correspondence to the experimental data. The full ensemble size is reached when no further improvement can be achieved by the addition of conformers. CoNSEnsX${}^{+}$ uses the SHIFTX program [28] to calculate chemical shifts and PALES [29] to estimate RDCs from the structures. We have introduced the calculation of secondary chemical shifts using the neighbor-dependent random coil values reported in [30]. The default Karplus equation parametrization was used to derive J-couplings [31].

The selected ensembles were also analyzed using CoNSEnsX${}^{+}$ and were compared with principal component analysis as implemented in the ProDy package [32].

### 2.2. Molecular Dynamics Simulations

A 1 μs all-atom simulation on Cd3ϵ in an explicit SPC/E water model [33] was performed with GROMACS (version 2020) [20] using the Amber ff99SB [34] forcefield. After neutralization and a short energy minimization, the production run was preceded by 1 ns NVT and NPT equilibrations. The simulation was run at 300 K using GPU acceleration. Structures taken at every 50 ps were used to obtain an ensemble with 20,001 conformers. All structures were retained in order to not reduce conformational variability by omitting the more extended structures at the start of the simulation.

## 3. Results

### 3.1. Implementation of the DIPEND Pipeline

DIPEND is implemented in Python3 to offer high flexibility and adaptability to specific needs. The program is running on a standard personal computer using one thread, which costs about 17 h for generating 1000 structures for a 50 amino acid protein. For about 100 amino acids, for 1000 structures, it costs around 190 h (about a week). In brief, the program performs the following main steps:

- Build an initial extended structure (invokes ChimeraX);
- Select the dihedral angles of each residue according to the distribution settings (see Methods);
- Set the dihedral angles to the selected values (invokes ChimeraX);
- Preliminary check for CA-CA clashes to filter out largely unrealistic structures (invokes an in-house C++ program);
- For clashing structures, an “unknotting” attempt can be performed if chosen with the options;
- Optimize side chains (invokes Scwrl4);
- Short energy minimization (invokes GROMACS);
- All-atom clash check (invokes ChimeraX).

DIPEND is implemented in Python3 with a small part in C++ and is freely available on GitHub https://github.com/PPKE-Bioinf/DIPEND. It requires ChimeraX and Scwrl4 as well as GROMACS for the optional but recommended optimization step. As the neighbor-dependent Ramachandran probabilities are provided on a grid with 5-degree resolution, this is also the resolution of the dihedral angles used to build the models. Thus, the GROMACS optimization step is necessary to relax this constraint.

The only input without a predefined default value is the sequence of the polypeptide to be built. The possible parameter settings are detailed in the Methods section.

For segments or individual amino acid residues, additional Ramachandran probability distributions can be provided. The easiest way to do this is to specify a ϕ,ψ dihedral angle pair and an associated standard deviation, for which the program generates a 2D normal distribution on the Ramachandran map. This distribution then can be combined with the neighbor-dependent ones using a user-defined weighting. Utilizing this feature, local structural preferences for selected amino acids can be shifted. A different distribution + weighting can be specified for each residue if desired.

### 3.2. Addition of Secondary Chemical Shift Analysis to the CoNSEnsX${}^{+}$ Server

To facilitate the analysis of local structural preferences in more detail, we have upgraded our CoNSensX${}^{+}$ web service with the analysis of secondary chemical shifts and made the reporting of these the default, while keeping the original chemical shift analysis results accessible. For secondary chemical shifts, we have used the neighbor-corrected values reported by [30]. The up-to-date version of CoNSEnsX${}^{+}$ is available on GitHub https://github.com/PPKE-Bioinf/consensx.itk.ppke.hu and also in dockerized form.

### 3.3. Overview of the DIPEND-Generated Ensembles

To test the pipeline, we have generated ensembles for two selected protein segments for which experimental data are available. The ensembles were analyzed using CoNSEnsX${}^{+}$, and we have also selected subensembles using one or more parameters. We have generally used RMSD as a measure to be improved during selection. For chemical shifts, RMSD is the same value irrespective of whether the full or the secondary shifts are analyzed. As the secondary chemical shifts are usually very small for disordered regions, a selection based on one atom type typically does not result in considerable improvement in other shift types in terms of correlation. This is somewhat surprising as the secondary shifts in principle report on the same structural feature, but our observation emphasizes the sensitive nature of such shifts to subtle local structural differences, especially when the secondary shifts are small.

### 3.4. Analysis of the Cd3ϵ Disordered Cytoplasmic Segment

After inspection of the secondary chemical shifts, we decided to omit the chemical shifts of the last residues of the segment Ile57 as an outlier because all of its secondary shift values were about two times larger than observed for all other residues. Based on several preliminary runs, a slight additional distribution was set up for the segment between residues 25–35 to enhance its extended nature using the dihedral angles −120,120 with a standard deviation of 10 degrees and a weight of 0.4. The generated ensemble contained 5000 conformers.

For Cd3ϵ, the generated ensemble already shows relatively low RMSD values between the observed and calculated chemical shifts, and the correlation between the full chemical shifts is excellent, consistent with a largely random disordered structure (Table 1). The correspondence to the secondary shifts can still be increased with selection, and a very low RMSD and high correlation can be achieved if selecting for CA chemical shifts only. In this case, the correlation to CB chemical shifts increases slightly but remains poor. Using both CA and CB shifts, a larger ensemble is selected with lower but acceptable correlation for both shift types, indicating that trends in local conformation along the sequence are acceptably well reproduced.

**Figure 2:**
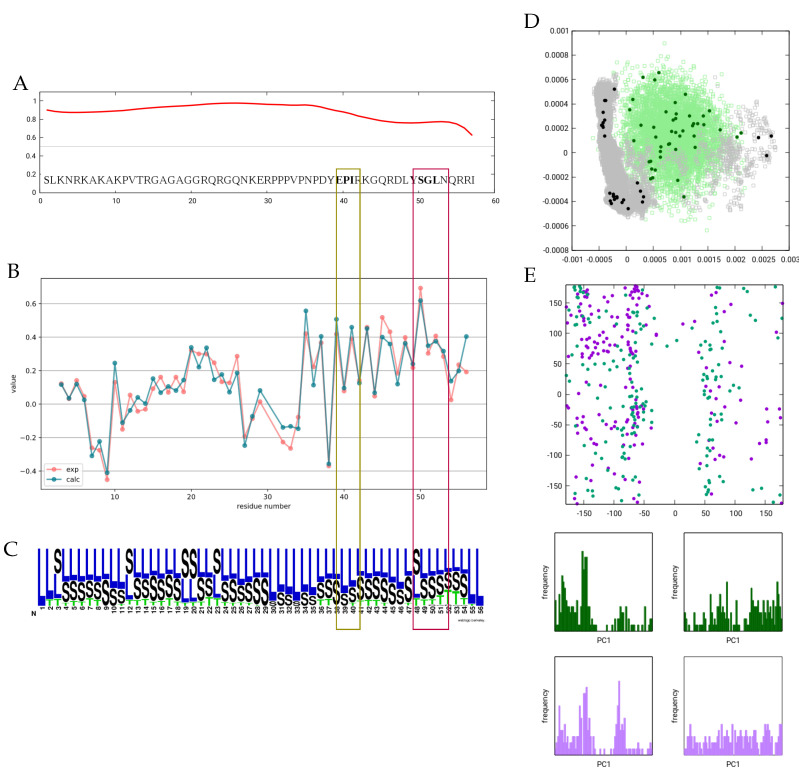
Overview of the Cd3ϵ ensemble. (**A**) Disorder propensity as predicted by the IUPred3 server. (**B**) Observed and calculated CA secondary chemical shifts. (**C**) Secondary structure logo for the ensemble selected based on CA chemical shifts. DSSPcont states are averaged for all models. Figure prepared with Weblogo. (**D**) PCA of the simulated, generated and selected (sub)ensembles. (**E**) Ramachandran plot of all residues in the ITAM1 (dark-green) and ITAM2 (purple) motifs in all structures of the selected ensemble.

We have also performed a selection from a set of conformers generated by a 1 μs all-atom molecular dynamics simulation in explicit water. The subensemble obtained from this selection exhibits considerably worse correspondence to experimental data than the one selected from the DIPEND-generated pool. During the MD simulation, Cd3ϵ quickly adopts a compact structure, and principal component analysis reveals that the selected subensemble samples this compact state as well as several frames from the extended conformation close to the initial state. In contrast, the DIPEND-generated structures provide a more even sampling of the conformational space, and the selected conformers are also more evenly distributed (Figure 2). This latter scenario is more in line with the expected behavior of an unfolded ensemble, but more independent parameters would be needed to confirm the accuracy of the DIPEND-selected ensemble model of Cd3ϵ conformations.

It was previously noted that the ITAM motifs exhibit a slight helical propensity, which is reflected in the CA secondary chemical shifts. In our ensemble, this propensity is not sufficient to cause the emergence of any DSSP ‘H’ states, and only a slight increase near the second motif is observed. The rationale for this observation can be that for a hydrogen bond to occur and thus be recognized as ‘T’ by DSSP, four consecutive residues should be simultaneously in a helical conformation, and for state ’H’, consecutive hydrogen bonds should be present. In a highly dynamic molecule with such small secondary chemical shifts, this occurs with only a very low probability. The Ramachandran plot of the 2 × 4 residues in the two ITAM motifs also does not reveal enrichment of backbone torsions in the α-helical region, although a slight preference for the ϕ torsion near −54 degrees can be observed.

### 3.5. The Single α-Helical Segment of Myosin VI

Using the published supplementary information, we have extended the BMRB entry 30591 [26] with N-H, N-C and C-H RDC data; ${}^{3}J_{HNHA}$ scalar couplings; and $S^{2}$ order parameters. In our selection, we have consistently used RMSD as a target function. It should be noted that in the case of RDCs, we deliberately do not intend to make any direct comparison with the Q-values reported by Barnes et al. because our calculation methodology differs. First, they used scaled RDCs to consider the dynamics of the molecule yet fitted the values to a highly rigid structure. Second, they used an alternative formula derived from the alignment tensor in the denominator to obtain the Q-values to account for the uneven sampling of the orientations by this rod-like structure. Third, our calculation method assumes an independent and different orientation of the members of the ensemble, each of which is separately fit to obtain the best correspondence with experimental values before averaging the RDCs for the ensemble. We have chosen this approach by reasoning that the changes in the molecular shape, i.e., the deviations from the straight rod-like structure are expected to influence the alignment substantially for the individual conformers. Thus, there is no universal alignment tensor for all the structures considered in our calculations.

For this highly α-helical segment it is evident that the helical region of the Ramachandran map should be sampled with high probability. Therefore, we have chosen to use an additional distribution to bias conformational sampling towards helical regions. However, analyzing the secondary chemical shifts, it is expected that the helical propensity of the molecule decreases more substantially and in a longer segment at the C-terminus. To account for this effect, we built our model using two additional distributions, both centered at the dihedral angles −58, −47, and having a standard deviation of 10 degrees, but for residues 1–55, this was applied with a weight of 0.99, whereas for residues 56–68, it was applied with a weight of 0.80.

A pool of 5000 structures was generated with these parameters, and then various selection settings were tested with CoNSEnsX${}^{+}$. Below, we report the results obtained with selection from the RMSD as a target measure and using the following relative weights: CA chemical shifts, 10, ${}^{3}J_{HNHA}$ scalar couplings, 5, N-H, N-C and C-H RDCs (measured in 100 mM NaCl), 1.

**Figure 3:**
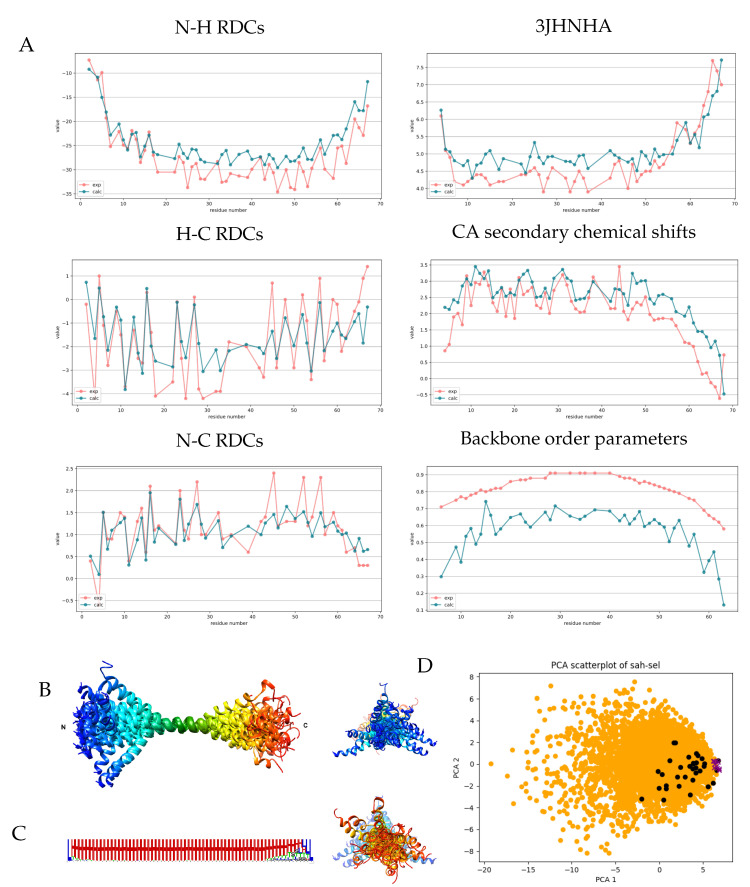
Measured and back-calculated NMR parameters and structural characteristics for the selected SAH ensemble. (**A**) Measured (red) and calculated (blue) values of some NMR parameters for the selected SAH ensemble. Calculated values were obtained with CoNSensX+. (**B**) Ribbon representation of the selected 37 conformers of the MYO VI SAH domain, superimposed for residues 28–42. Rainbow coloring from N to C terminus. Figure prepared with UCSF Chimera. (**C**) Secondary structure logo generated from averaging all DSSP state probabilities calculated with DSSPcont for all 37 models. Figure prepared with Weblogo. (**D**) PCA plot showing the distribution along modes 1–2 of the generated 5000 (orange), the selected 37 (black) structures and the 10 deposited conformers in PDB entry 6OBI (purple). Mode 1 corresponds to the end-to-end distance of the structures.

The selected ensemble shows improvement for all parameters considered and even for the CB shifts not included in the selection (Table 2). As expected, the structures show deviation from a straight α-helix. In Figure 3, the structures are shown superimposed through residues 28-42, corresponding to the most rigid segment identified by Barnes et al. based on NMR relaxation data. It should be noted that in the case of such a rod-like structure, the visualization highly depends on the segment chosen for superposition. DSSPcont analysis shows that the helical secondary structure is dominant throughout the sequence. A small fraction of turns (DSSP state T) is present along the full sequence, indicating occasional interruption of the hydrogen-bond pattern and accounting for the kinks observed in the structure. Helicity decreases at the C-terminal region from around residue 55, which is in agreement with the secondary chemical shifts and our applied biased distribution influencing local structural preferences. Investigating the relationship between the fully generated ensemble and the selected one using principal component analysis reveals that only a limited region of the conformational space explored is represented by the selected conformers, indicating that adjusting the local structural preferences was necessary to obtain the set of structures corresponding to the experimental parameters, but alone, it is not sufficient. The most important question is how well our selected ensemble actually describes the internal motions of the Myosin VI SAH segment, especially in relation to the deposited structure which was refined against a set of NMR observables overlapping with those used here. Again it should be noted that for the 6OBI structure, it was not a goal to reflect structural heterogeneity at the level of the ensemble, and the internal dynamics was taken into account by scaling the RDC parameters used in the structure calculation. We have used the unscaled values and explicitly selected our ensemble for secondary CA chemical shifts and ${}^{3}J_{HNHA}$ scalar couplings, whereas no NOE restraints were used. In addition, our selected ensemble contains 37 conformers compared to the 10 models in 6OBI, and averaging the parameters over a larger ensemble renders achieving correspondence to experimental data easier. Perhaps our most interesting observation is that the correlation to the backbone $S^{2}$ order parameters is excellent for our ensemble, although the calculated values are considerably lower than the experimental ones. It should be noted here that for this rod-like structure, the structure-based estimation of order parameters [35] is heavily dependent on the exact mode of superposition of the structures. For this calculation, we have chosen to use all residues. In summary, we believe that our ensemble successfully recapitulates the nature of the structural rearrangements in the SAH segments, meaning that for most of the region, local disruptions in the helical structure are prevalent, leading to occasional kinks in the structure, and partial unfolding of the helix occurs only at the 10–15 C-terminal residues. Barnes et al. concluded that $S^{2}$ order parameters and RDCs report on the same kinds of motions, which is—if we accept the validity of the correlation to the $S^{2}$ values—recapitulated by our ensemble but indicates that faster motions might be of smaller amplitude, accounting for the high experimental $S^{2}$ order parameters and the magnitude difference with the calculated ones in our ensemble generated using RDC data.

## 4. Discussion

We have developed an open-source pipeline, DIPEND, that can be used to generate conformer pools to model ensembles of intrinsically disordered proteins. Given the nature of the choice of the dihedral angles, the probability of steric clashes increases with the size of the protein segment, and as a result, the computation time increases. The pipeline can handle protein segments up to 100 amino acids at a reasonable CPU time on a single thread without the “unknotting” step. Naturally, the program can easily be run in multiple instances to make use of today’s common multicore CPUs, pushing the practical limit further. Our experience shows that switching on the unknotting step is advised for sequences longer than 75 residues, as for shorter segments, it does not necessarily provide an advantage over the complete resampling of all dihedrals.

DIPEND itself performs only the generation of the structures; thus, any kind of selection/analysis approach or tool, such as CoNSEnsX${}^{+}$ or BME [36], can be used to refine the model. The detachment of ensemble generation from the analysis has both advantages and disadvantages. The upside is that the generation can be adjusted precisely to the given system, and even multiple tools (such as classical MD simulation) can be used to extend the ensemble before analysis. The downside is that in this way, separate tools have to be used for subsequent steps, the compatibility of which might not be straightforward. For example, the atomic nomenclature of an MD-derived ensemble might not necessarily match that used in a BMRB entry. To help with such issues, a number of small conversion scripts are already available on the CoNSEnsX${}^{+}$ page. In summary, we believe that for research purposes, the transparency and flexibility of the DIPEND pipeline outweigh the potential disadvantages listed above.

Conformational sampling is one of the main technical issues that has to be solved in modeling protein flexibility and dynamics. The ideal tool would sample only a region around the biologically relevant one, but with good internal spacing. The problem is that this region is not known a priori, and, especially for intrinsically disordered proteins, the number of accessible conformations with potential relevance is astronomical. Fully randomized approaches to generate a large conformer pool can in principle provide suitable states but, depending on the system, only with low probabilities. The conventional solution is to generate a large ensemble and then use selection/reweighting to obtain the biologically relevant subspace [37]. The subtle preferences reflected by secondary chemical shifts, however, can be not trivially captured from an unbiased ensemble. DIPEND intends to overcome this difficulty by applying a neighbor-dependent sampling of the Ramachandran map and the possibility of adding a biasing distribution that can be used to avoid conformations that are not characteristic for a given region of a molecule and/or to enhance sampling or regions where a priori knowledge or exploratory calculations indicate the need for this.

Selection-based ensemble generation will only be as good as the initial pool of conformations, and naturally, the availability and nature of different structural parameters will greatly affect the outcome. DIPEND is designed to provide a means to generate an initial ensemble that covers the functionally relevant region of the conformational space of a protein segment, providing a suitable input for different selection approaches.

## Figures and Tables

**Table 1:** RMSD and correlation of the back-calculated chemical shifts of the generated ensembles of the Cd3ϵ segment.

	Generated 5000		CA+CB rmsd		CA rmsd		Selected from MD	
			Selected (84 Models)		Selected (45 Models)		(CA rmsd, 29 Models)	
	rmsd	corr.	rmsd	corr.	rmsd	corr.	rmsd	corr.
CA full	0.458	0.996	0.211	0.999	0.073	1.000	0.400	0.997
CA secondary		0.296		0.696		0.953		0.451
CB full	0.550	0.999	0.137	1.000	0.601	0.998	0.831	0.997
CB secondary		0.233		0.764		0.322		0.301

**Table 2:** RMSD and correlation of selected back-calculated NMR parameters of MYO VI SAH ensembles.

	6OBI (10 Models)		Generated 5000		Selected (37 Models)	
	rmsd	corr.	rmsd	corr.	rmsd	corr.
N-H RDC	4.021	0.746	7.304	0.756	3.676	0.917
H-C RDC	1.709	0.456	1.440	0.671	1.058	0.807
N-C RDC	0.822	0.118	0.541	0.576	0.351	0.838
3JHNHA	0.602	0.789	0.765	0.625	0.518	0.903
CA secondary	0.969	0.712	0.866	0.705	0.688	0.903
CB secondary	0.915	0.389	0.967	0.561	0.970	0.587
N-H S2	0.201	0.488			0.258	0.871

## Data Availability

The datasets supporting the conclusions of this article are included within the article and the supplementary file. The supplementary file DIPEND_Supplementary.zip contains the input parameter files (parameters.in, distributions.in) for Dipend.py used to generate the ensembles described in the manuscript, the modified NMR-STAR files used for the CoNSEnsX${}^{+}$-based analysis and selection and a PDB file with a selected ensemble for CD3e (selection based on CA shifts only) and Myo VI SAH (selection based on all parameters described in the manuscript) in separate directories.

## Supplementary Materials

The following are available at https://www.mdpi.com/article/10.3390/biom11101505/s1, Data set S1: input parameter files (parameters.in, distributions.in) for Dipend.py used to generate the ensembles described in the manuscript, the modified NMR-STAR files used for the CoNSEnsX+-based analysis and selection as well as a PDB file with a selected ensemble for CD3e (selection based on CA shifts only) and Myo VI SAH (selection based on all parameters described in the manuscript) in separate directories

## Abbreviations

The following abbreviations are used in this manuscript:

BMRB	Biological Magnetic Resonance Data Bank
GUI	Graphical User Interface
IDP	Intrinsically Disordered Protein
NMR	Nuclear Magnetic Resonance
NOE	Nuclear Overhauser Effect
MD	Molecular Dynamics
PDB	Protein Databank
PCA	Principal Component Analysis
RDC	Residual Dipolar Coupling
RMSD	Root Mean Square Deviation
